# Extensive mitochondrial gene rearrangements in Ctenophora: insights from benthic Platyctenida

**DOI:** 10.1186/s12862-018-1186-1

**Published:** 2018-04-27

**Authors:** Hanan Arafat, Ada Alamaru, Carmela Gissi, Dorothée Huchon

**Affiliations:** 1 School of Zoology, Tel-Aviv University, Tel-Aviv, Israel; 2 Department of Biosciences, Biotechnology and Biopharmaceutics, University of Bari “Aldo Moro”, Bari, Italy; 3 IBIOM, Istituto di Biomembrane, Bioenergetica e Biotecnologie Molecolari, CNR (Italy), Bari, Italy; 4 The Steinhardt Museum of Natural History and National Research Center, Tel-Aviv University, Tel-Aviv, Israel

**Keywords:** *Coeloplana*, *Vallicula*, mtDNA, Gene loss, Extreme evolutionary rate

## Abstract

**Background:**

Complete mitochondrial (mt) genomes have been sequenced for thousands of animals and represent a molecule of choice for many evolutionary studies. Nevertheless, some animal groups have remained under-sampled. Ctenophora (comb jellies) is one such example, with only two complete mt sequences determined hitherto for this phylum, which encompasses ca. 150–200 described species. This lack of data derives from the extremely fast mt evolutionary rate in this lineage, complicating primer design and DNA amplification. Indeed, in the two ctenophore mt genomes sequenced to date, i.e. those of *Mnemiopsis leidyi* (order Lobata) and *Pleurobrachia bachei* (order Cydippida), both rRNA and protein coding genes exhibit an extraordinary size reduction and have highly derived sequences. Additionally, all tRNAs, and the *atp6* and *atp8* genes are absent. In order to determine whether these characteristics are shared by other ctenophores, we obtained the complete mt genomes of three benthic ctenophores belonging to the so far unsampled order of Platyctenida: *Coeloplana loyai, Coeloplana yulianicorum* and *Vallicula multiformis*.

**Results:**

The mt genomes of benthic ctenophores reveal the same peculiarities found in *Mnemiopsis* and *Pleurobrachia*, demonstrating that the fast evolutionary rate is a general trait of the ctenophore mt genomes. Our results also indicate that this high evolutionary rate not only affects the nucleotide substitution but also gene rearrangements. Indeed, gene order was highly rearranged among representatives of the different taxonomic orders in which it was close to random, but also quite variable within Platyctenida, in which the genera *Coeloplana* and *Vallicula* share only four conserved synteny blocks. However, the two congeneric *Coeloplana* species display exactly the same gene order. Because of the extreme evolutionary rate, our phylogenetic analyses were unable to resolve the phylogenetic position of ctenophores within metazoans or the relationships among the different Ctenophora orders. Comparative sequence-analyses allowed us to correct the annotation of the *Pleurobrachia* mt genome, confirming the absence of tRNAs, the presence of both rRNA genes, and the existence of a reassignment of codon TGA from tryptophan to serine for this species.

**Conclusions:**

Since Platyctenida is an early diverging lineage among Ctenophora, our findings suggest that the mt traits described above are ancestral characteristics of this phylum.

**Electronic supplementary material:**

The online version of this article (10.1186/s12862-018-1186-1) contains supplementary material, which is available to authorized users.

## Background

The typical metazoan mitochondrial (mt) genome is a small (~ 20 kb), circular DNA molecule containing 37 genes – 13 protein coding genes (the cytochrome oxidase 1–3 [*cox1–3*]; the ATPase subunit 6 and 8 [*atp6/8*]; the cytochrome b [*cob*]; the NADH dehydrogenase subunit 1–6 [*nad1–6*]), 22 transfer RNA (tRNAs) genes and 2 ribosomal RNA (rRNA) genes (the small subunit ribosomal RNA [*rns*] and the large subunit ribosomal RNA [*rnl*]) [1, 2]. This compact molecule possesses several characteristics that have made it a perfect sequencing target. First, mt genomes are present in high copy numbers [3], since eukaryotic cells usually contain several mitochondria and each mitochondrion possesses numerous copies of the mt genome. Second, mt genomes are extremely compact – most are intron-less and non-coding regions are extremely small [3]. Consequently, it is usually easier to amplify and sequence mt genes than nuclear ones. Finally, the same set of orthologous single-copy genes is conserved in most animal taxa, thus facilitating sequence comparisons and, above all, allowing the reconstruction of gene trees that correspond to species trees [4].

The above-noted features have made the mt genome a molecule of choice for many fields of genomics and molecular evolution. For example, complete mt genome sequences have been used to resolve the phylogenetic relationships of many animal groups [5], to study the transfer of organelle genes to the nucleus [6] and to study the evolution of gene rearrangement [7]. Additionally, the mt *cox1* gene is used as a universal “DNA barcode” [8] for animals and, consequently, for a given species, a complete mt genome is often sequenced from one individual as a prerequisite of population studies [9].

One direct consequence of the high interest in mt genomes is the large and increasing number of complete mt genomes available in public databases (i.e., over 7300 complete mt genomes of animals are available at the National Center for Biotechnology Information [NCBI] - https://www.ncbi.nlm.nih.gov/genome/browse/) [3], and numerous databases and web servers are dedicated to their annotations and comparisons [10, 11]. However, some animal phyla, such as Ctenophora, are under-represented in these databases [12].

Ctenophora (comb jellies) comprise a rather small phylum of marine animals that encompasses about 150–200 species [13]. Most ctenophores are planktonic predators with a gelatinous body similar to jellyfish (Cnidaria, Medusozoa), for which they are often mistaken. Unlike jellyfish and other cnidarians, ctenophores lack stinging cells and are characterized by the presence of comb rows composed of cilia, which are used for swimming, and tentacles, which possess a special cell type, the colloblasts, used for grasping and feeding. Ctenophores are traditionally divided into two classes, Tentaculata and Nuda, based on the presence or absence of tentacles throughout their lives, respectively [13]. However, molecular analyses suggest that Tentaculata, which include eight orders, is paraphyletic, and that the single Nuda order – Beroida – is nested within Tentaculata [14–17]. Among ctenophores, Platyctenida is the only order that includes benthic species. Most members of the Platyctenida do not possess comb rows in the adult stage and externally resemble flat worms, except for the presence of two long retractile tentacles. Interestingly, molecular studies suggest that Platyctenida is not the first ctenophore lineage to diverge, and that the adaptation to a benthic environment is a derived characteristic [14–17].

To date, only two complete mt genomes have been sequenced in Ctenophora: the genome of *Mnemiopsis leidyi* (order Lobata) [12] and that of *Pleurobrachia bachei* (order Cydippida) [18]. Both these genomes strongly diverge from the “typical” mt genome described above (see Lavrov and Pett [1]). Indeed, they are of very small size (10–11 kilobases [kb]); lack the *atp8*, *atp6* and most/all tRNA genes; demonstrate a length reduction; and a high sequence divergence in both protein coding and rRNA genes. These mt genomes thus reveal an extremely high evolutionary rate. It is unclear, however, whether all these features are shared by all ctenophores or only by the few analysed lineages to date.

Here, we report the sequencing and analysis of the complete mt genomes of three benthic ctenophore species belonging to the order Platyctenida and the family Coeloplanidae: *Coeloplana loyai, Coeloplana yulianicorum* and *Vallicula multiformis*. The analyses of these new species have enabled us to amend the annotation of the *Pleurobrachia* mt genome and to confirm a modification of the genetic code in the latter.

## Results

### General features of the Coeloplanidae mt genomes

**Fig. 1:**
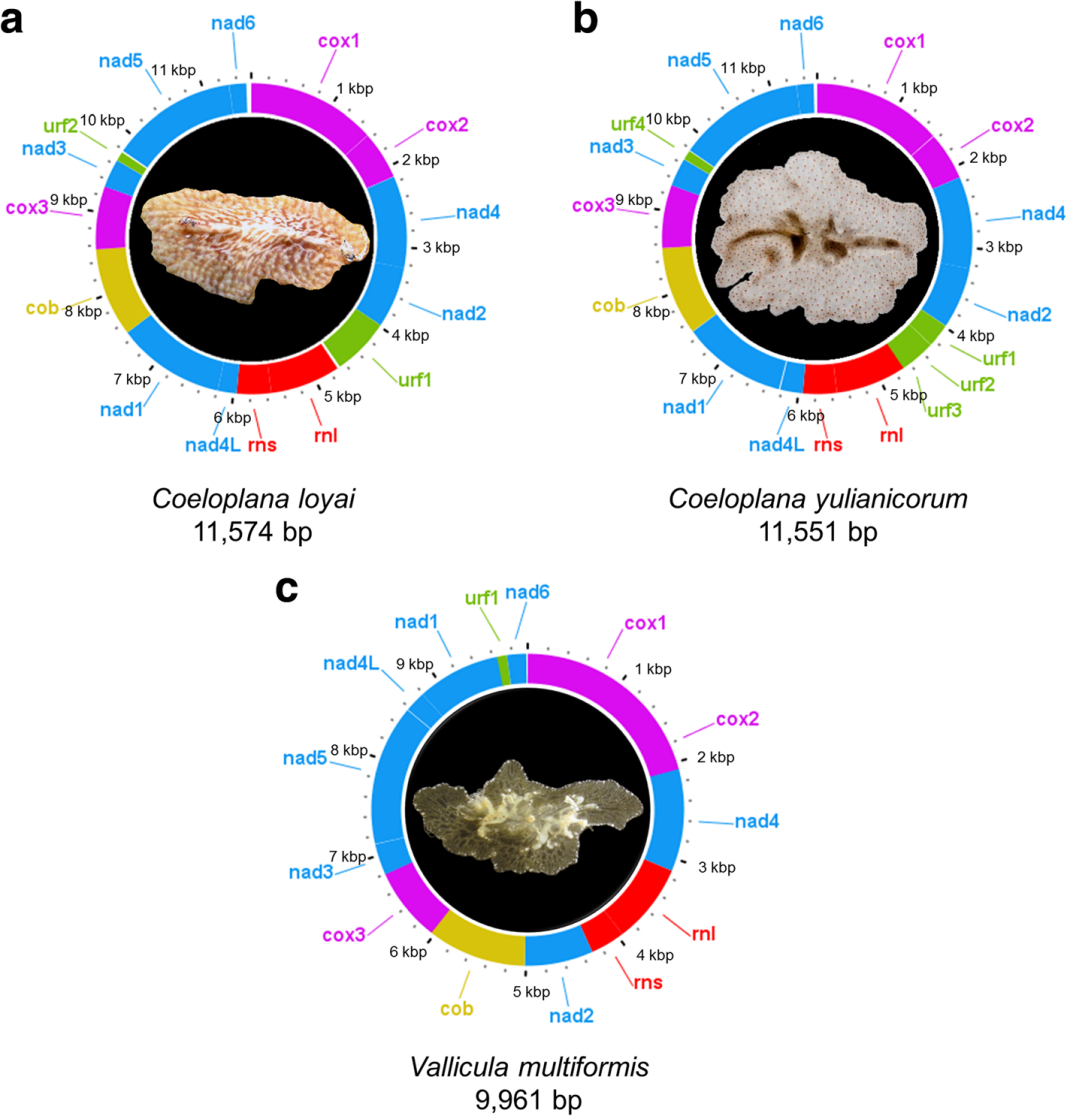
Organization of the Coeloplanidae mt genomes. **a** *C. loyai*. **b** *C. yulianicorum*. **c** *V. multiformis*. All genes are transcribed clockwise. Large and small ribosomal RNA subunits are marked in red. The protein coding genes are indicated by different colours: in purple - the cytochrome c oxidase genes (Complex VI), in blue - NADH dehydrogenase genes (Complex I), in yellow - the cytochrome b (complex III). Green areas are URFs (unidentified reading frames). Finally, noncoding regions are in white. A photo of each organism is provided in the centre of the corresponding mt genome

The circular mt genomes of *C. loyai*, *C. yulianicorum* and *V. multiformis* are 11,574, 11,551, and 9961 base pairs (bp) long, respectively. The three sequenced species contain the same set of genes: 11 protein coding genes (all those present in typical animal mt genomes, except for *atp6* and *atp8*) and 2 rRNAs (*rnl* and *rns*). In addition, from 1 to 4 unassigned ORFs (Unidentified Reading Frames, named URFs 1–4), ranging from 102 to 687 bp, were identified in each species (Fig. 1). All annotated URFs were predicted to encompass from 1 to 5 putative transmembrane domains. All these genes are encoded on the same strand (Fig. 1).

No tRNAs were identified. Indeed, all putative tRNAs detected by the three tRNA annotation software programs (see Methods) were located within ORFs and possessed a structure that did not fit the canonical mt tRNA structure [19]. They were thus considered to be false positive hits.

**Fig. 2:**
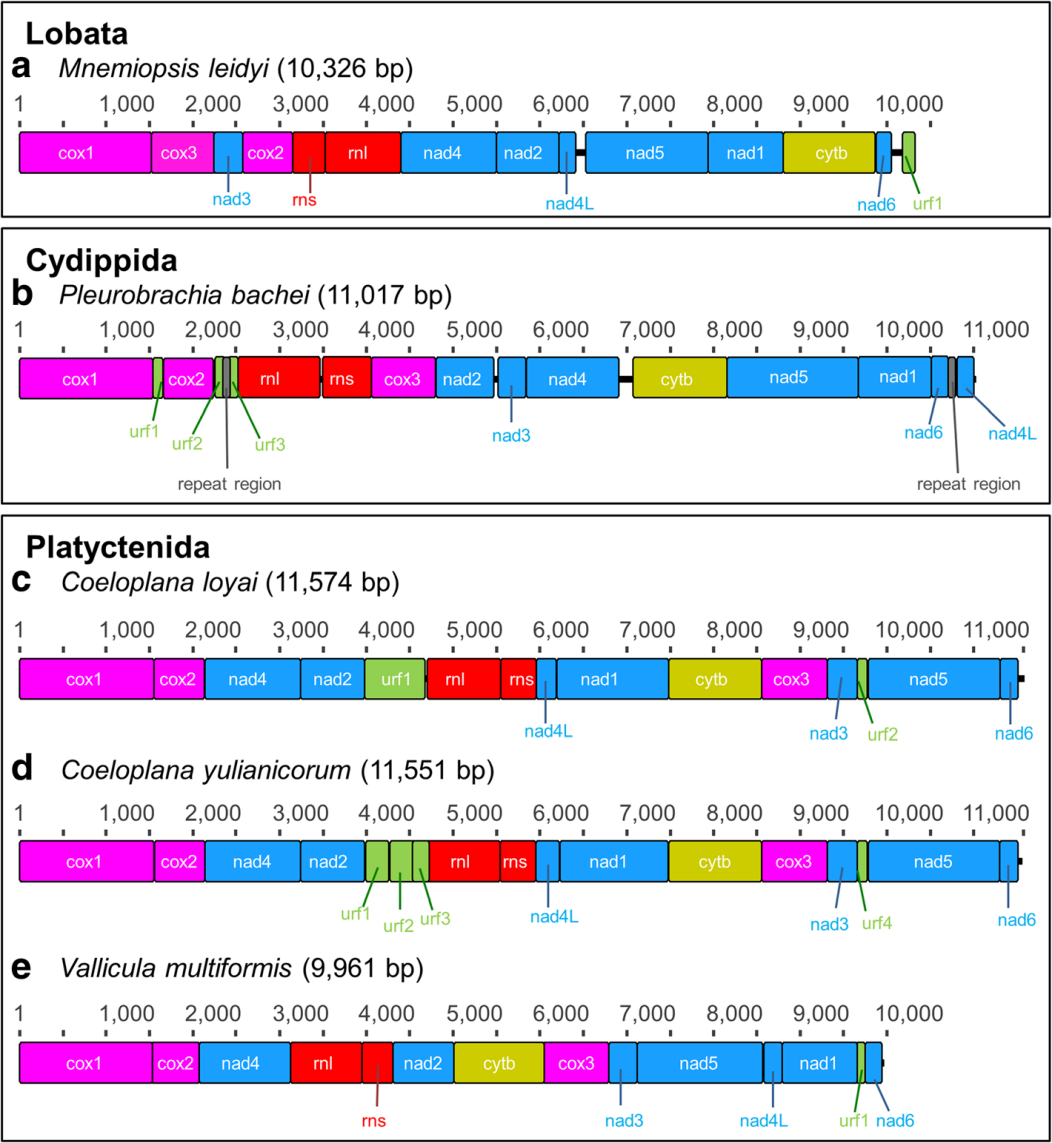
Comparison of mt gene order between Ctenophora orders. **a** *M. leidyi*. **b** *P. bachei*. **c** *C. loyai*. **d** *C. yulianicorum*. **e** *V. multiformis*. The genome of *Pleurobrachia bachei* has been re-annotated. Each orthologous protein coding or rRNA gene is indicated by a different colour. Black connecting lines between genes indicate noncoding regions. URFs are indicated in green. Remarkably, no syntenic regions were shared by all ctenophore species

While gene order is conserved between the mt sequences of the two *Coeloplana* species, only four synteny blocks are conserved between the two genera *Coeloplana* and *Vallicula* (*cox1-cox2-nad4*, *nad4L-nad1*, *cob-cox3-nad3*, and *rnl*-*rns*) (Fig. 2). The gene order is thus conserved at the congeneric level but already quite variable at the intra-family level. Considering the other ctenophore taxonomic orders, only one synteny block is conserved (*cox3-nd3*) between Platyctenida and the Lobata *M. leidyi*, and no synteny blocks are shared between the Cydippida *P. bachei* (re-annotated, see below) and the other ctenophore species. The results of the breakpoint analyses support these observations (see Table 1, where values of the normalized breakpoint distances - BDn - close to one indicate extreme rearrangements close to random). Indeed, the BDn computed among Ctenophora orders range between 0.69 and 0.92.

**Table 1:** Breakpoint distances computed for each pair of ctenophore mt genomes

	*C. loyai*	*C. yulianicorum*	*V. multiformis*	*M. leidyi*	*P. bachei*
*Coeloplana loyai*	–	0	5	9	11
*Coeloplana yulianicorum*	0	–	5	9	11
*Vallicula multiformis*	0.38	0.38	–	11	12
*Mnemiopsis leidyi*	0.69	0.69	0.85	–	12
*Pleurobrachia bachei*	0.85	0.85	0.77	0.92	–

Breakpoint distances [38] and normalized breakpoint distances are given above and below the diagonal respectively

Concerning base composition, the mt genomes of *C. loyai*, *C. yulianicorum* and *V. multiformis* are extremely AT rich, with AT contents of 84, 82.9 and 84.4%, respectively. The most frequent nucleotide in those genomes is T, which represents more than 50% of the nucleotide in all three species (60.2–60.6%).

No repeated region longer than 50 bp was found in these mt genomes. The longest repeated elements (31–21 bp) found in each species were always located in low complexity regions and composed mainly of poly-T repeats.

### Re-annotation of the *Pleurobrachia bachei* mt genome

Analysis of the alignments of the mitochondrial protein genes led us to discover several errors in the original annotation of the *Pleurobrachia* mt genome (accession JN392469 [18]) (see Additional file 1). For example, we found that the *nad4L* gene had been erroneously annotated as *nad3*. The true *nad3* was not identified by Kohn et al. [18] but included as part of the *nad4* gene. Indeed, in *Pleurobrachia*, but not in other ctenophores, the *nad3* gene shares the same translation frame as the downstream *nad4* gene and possesses an incomplete stop codon. Similarly, the *rns* gene was originally annotated as *rnl* and the two tRNAs identified by Kohn et al. [18] were found in the region that we now recognize as the *rnl*. In other words, the two tRNAs annotated by Kohn et al. [18] are now considered as being only tRNA-like structures (Additional file 1). Our amended annotation thus confirms the absence of tRNA genes and the presence of *rns* and *rnl* in the *P. bachei* mt genome. Moreover, we definitively identified the *nad2* and *nad6* genes in two regions not corresponding to those suggested by Kohn et al. [18] (Additional file 1).

The protein gene alignments also indicated that the 5′ region of the *cox1* gene was truncated in *P. bachei*, since it lacks 25 amino acid positions highly conserved in the remaining ctenophores (Additional file 2). The region upstream to the *cox1* gene includes several poly-T, and thus, we suspected the introduction of a frame shift due to erroneous sequencing of the number of T in these homopolymers by Kohn et al. [18]. To verify this, we downloaded Illumina reads used for assembling the *P. bachei* nuclear genome (run SRR1174875 [14] of the Sequence Read Archive of NCBI - https://trace.ncbi.nlm.nih.gov/Traces/sra/) and mapped them against the mt sequence of *P. bachei* (accession JN392469). This alignment confirmed that a T is missing eight bp upstream to the start of the *cox1* gene as defined by Kohn et al. [18]. The inclusion of this missing T extended the reading frame and enabled identification of the missing 5′ region of the *P. bachei cox1* (Additional file 2).

One and two URFs (length: 108–111 bp) were found upstream and downstream of *cox2* respectively, while a 84 bp long sequence was found to make up a perfect repeat about 8.2 kb apart from each other.

Finally, investigation of codon usage in conserved gene regions using GenDecoder v.1.6 [20] confirmed that in *P. bachei* the codon TGA, which codes for tryptophan in other ctenophores, is reassigned to serine as indicated by Pett and Lavrov [21]. An example of conserved serine positions coded by TGA codons in *P. bachei* is given in Additional file 2.

An updated annotation of the *P. bachei* genome is provided in Additional file 3.

### Protein-coding genes

Codon usage of the three benthic ctenophores *C. loyai*, *C. yulianicorum* and *V. multiformis* is provided in Additional file 4. Unlike for *P. bachei*, we did not detect any change in the mt genetic code of these species. In agreement with the observation that these sequences are AT-rich, the most abundant codons were TTT (Phe; 26.35, 26.29 and 26.54%, respectively), ATT (Ile; 8.72, 8.27, and 7.37%, respectively) and TTA (Leu; 9.74, 7.98 and 10.13%, respectively). Correspondingly, C- or G- rich codons demonstrated lower frequencies.

Concerning start codons, four start codons are used in Coeloplanidae (ATA, ATG, ATT, and TTA), with ATT and ATG being the favoured ones. In *C. loyai* and *C. yulianicorum* ATG is the most used (0.31 and 0.28% respectively), while ATT is the most abundant in *V. multiformis* (0.2%). In contrast, among canonical mt genes, TTA is only identified as a start codon of the *nad1* gene of *C. yulianicorum* and of the *nad2* gene of *P. bachei*. It is also the start codon of two of the URFs of *C. yulianicorum*. Because this codon is poorly used, it is thus possible that these URFs are in fact false positive. Concerning stop codons, both TAA and TAG are used in Ctenophora, with TAG being used only once in both *Coeloplana* species and in *Mnemiopsis*. Incomplete stop codons were only found in the *nad2* and *nad4L* of *V. multiformis*, the *nad3* of *P. bachei*.

### rRNA genes

The rRNA genes were identified in *C. loyai*, *C. yulianicorum* and in the re-annotated *P. bachei* using a secondary structure model (see Methods) based on *M. leidyi* rRNA sequences. This approach, however, failed to detect the *rns* gene in *V. multiformis* (i.e., no hits were obtained with an *E*-value < 0.1). The *rns* of *V. multiformis* was consequently detected using standard Blastn searches using the *rns* sequence of *Coeloplana* as query. The lengths of the rRNA genes in *C. loyai*, *C. yulianicorum*, *V. multiformis* and *P. bachei* were: 413, 408, 355 and 563 nt for the *rns*, respectively; 843, 847, 825 and 943 nt for the *rnl*. These values are similar to the rRNA lengths observed in *M. leidyi* (i.e., 368 and 878 nt for the *rns* and *rnl* respectively). Alignment of the rRNA sequences revealed that there are very few shared positions between the five ctenophore sequences, indicating an extremely fast evolutionary rate of the rRNA genes (Additional files 5 and 6). Accordingly, only a few of the helices observed in the rRNAs of *M. leidyi* [12] were conserved among ctenophores. Specifically, the *rnl* helices 73, 74, 89, 90, 92 and 93 (Additional file 5) and the *rns* helices 18, 28 and 44 (Additional file 6) were the only conserved helices among the five species. Indeed, in the region of helices 29, 30 and 43, the *rns* of *P. bachei* contains novel insertions that are not shared by the other ctenophores.

### Non-coding regions

Table 2 provides statistics on non-coding regions (NC) of the five ctenophores mt genomes. It should be noted that all ctenophore mt genomes (even *Mnemiopsis*) present URFs longer than 100 bp: according to the results of Blastn/Blastp against the NCBI nucleotide/protein databases, these URFs have no similarities to known peptides, so they could also be non-coding regions with accidental ORFs.

**Table 2:** Statistics on non-coding sequences (NC) in Ctenophora mitochondrial genomes

	Genome length (bp)	NC length (bp)		N° NC > 20 bp		Longest NC			
		with URFs	without URFs	with URFs	without URFs	with URFs		without URFs	
						bp	location	bp	location
*Coeloplana loyai*	11,574	930	132	4	2	687	*nad2-rnl*	59	*nad6-cox1*
*Coeloplana yulianicorum*	11,551	908	90	5	1	318	*nad2-rnl*	41	*nad6-cox1*
*Vallicula multiformis*	9961	119	25	1	0	94	*nad1-nad6*	13	*nad6-cox1*
*Mnemiopsis leidyi*	10,326	406	265	3	2	276	*nad6-cox1*	132	*nad6-urf1*
*Pleurobrachia bachei*	11,016	755	429	8	5	276	*cox2-rn*	161	*nad4-cytb*

When URFs are considered to be non-coding, *C. loyai* and *C. yulianicorum* possess the largest proportion of non-coding regions: 8 and 7.9%, respectively. In both these species, the longest non-coding regions are located between *nad2* and *rnl* and are 687 and 318 bp long, respectively. However, when URFs are treated as coding genes, *P. bachei* and *M. leidyi* possess the largest proportion of non-coding regions: 3.9 and 2.6% bp, respectively.

Among ctenophores, *V. multiformis* has the smallest number of non-coding regions: the total non-coding length is only 119 or 25 bp long, depending on whether URFs are included or excluded from non-coding, respectively (Table 2). The *V. multiformis* mt genome is consequently both the smallest ctenophore and metazoan mt genome reported to date (9961 bp long).

A correlation analysis indicates that the NC length strongly affects the overall size of the mt genome. In particular, plotting the mt genome size against the calculated NC lengths, we found the mt genome size to be significantly correlated to the NC length only when including URFs (*r* = 0.98).

### Extreme evolutionary rate of ctenophore mitochondrial genome

**Fig. 3:**
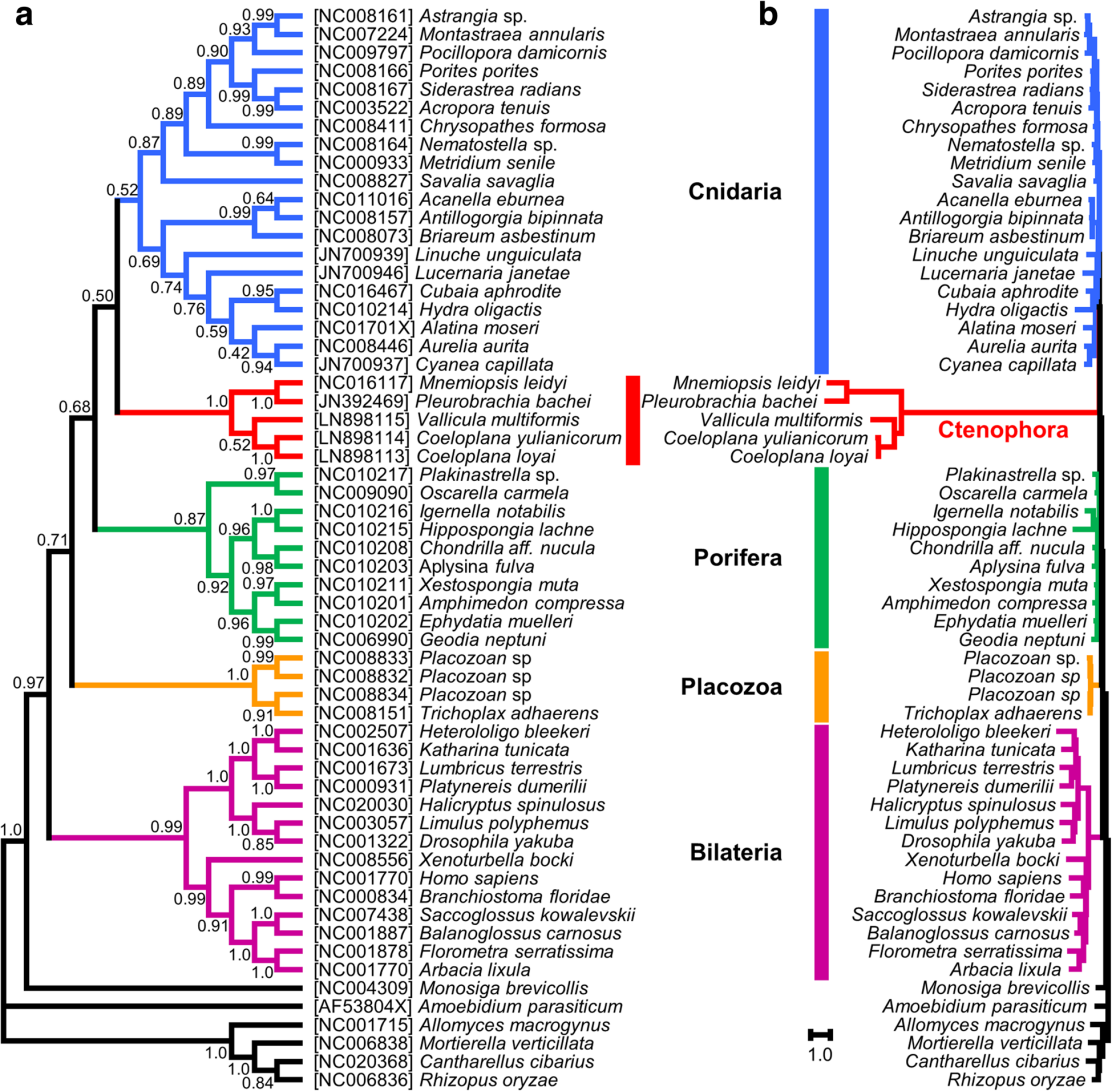
Phylogenetic position of Ctenophora within Metazoa using the most conserved mt genes. Bayesian tree reconstructed under the CAT+GTR + Γ mixture model from the seven most-conserved ctenophore genes (*cob*, *cox1–3*, *nad1*, *nad3* and *nad5*). **a** Topology and posterior support values. **b** Tree with branch length. The long ctenophore branches, in blue, exemplify the fast evolutionary rate observed in this phylum compared to all other animal groups

We performed phylogenetic analyses based on the protein sequences from the seven most conserved mt genes (*cob*, *cox1–3*, *nad1*, *nad3* and *nad5*), including species representatives of the main Metazoa phyla (see Methods). As exemplified in the reconstructed Bayesian phylogenetic tree (Fig. 3), all ctenophore mt genomes reveal very long branches and are therefore extremely fast evolving. Not only were ctenophores found to be distant from all other phyla considered but also between themselves. As a case in point, the branch length distance, as estimated in the phylogenetic tree, between the lobate *Mnemiospsis* and the benthic platyctenid *Vallicula* is far greater than the distance between human and sea urchin.

Because of the extreme mt evolutionary rates, the phylogenetic position of the ctenophores among animals is not resolved by the analyses of mt sequences (the 0.50 posterior probability (PP) support value is unreliable). Concerning the relationships among the analysed ctenophore orders, the small taxon sampling prevents conclusive observations. However, it should be noted that members of the Coeloplanidae have shorter branches compared to the branch leading to the cluster *Mnemiopsis* plus *Pleurobrachia* (indicating a lower evolutionary rate). Moreover, the monophyly of Coeloplanidae was surprisingly recovered with low support (PP = 0.52) while it received high or maximal support in previous molecular studies based on 18S rRNA [15] and transcriptome analyses [14].

## Discussion

The new mt sequences confirm that the key characteristics of the *Mnemiopsis* and *Pleurobrachia* mt genomes are also shared by benthic ctenophore species of the order Platyctenida. These characteristics include: the absence of *atp6*, *atp8* and all mt tRNA genes (Fig. 2); a strong reduction in the overall size and number of conserved helices in the mt rRNA genes (both *rns* and *rnl*); the presence of all genes on the same strand (Figs. 1 and 2); a base composition with a high AT%; and an extremely fast evolution rate (Fig. 3) [12, 18]. Most of these characteristics have led to a reduction in size of the ctenophore mt genome when compared to other animals. Indeed, it should be noted that the *V. multiformis* mt genome (9961 bp long) is the smallest animal mt genome sequenced to date.

Since Platyctenida are considered an earlier diverging lineage among Ctenophora than *Mnemiopsis* and *Pleurobrachia* [14, 16, 17], our findings suggest that the traits described above are ancestral in Ctenophora. However, the mt genome of representatives from the families Euplokamididae and Mertensiidae (order Cydippida), which were reported to diverge before Platyctenida [14, 16, 17], should be sequenced in order to confirm this contention.

The loss of mt-tRNA in ctenophores has been found to be correlated with gene losses in the nuclear genome. As a case-in-point, most of the mt aminoacyl tRNA synthetases (aaRS) – the enzymes that attach an aminoacid to their corresponding tRNA – are absent from the nuclear genomes of ctenophores [21]. There are two exceptions to the aaRS loss in Ctenophora: the mt-PheRS, which is assumed to be retained because of import constraints; and the mt-TrpRS, which is considered to be retained by genetic code constraint [1, 21, 22]. Specifically, the mt-TrpRS is assumed to be required for the aminoacylation of the mt-tRNATrp, which recognizes the TGA codon in the “Mold, Protozoan and Coelenterate” mitochondrial code. In *P. bachei*, however, where the TGA codon is reassigned to Ser, it has been shown that the mt-TrpRS has been lost [1, 21]. Our analyses did not indicate any mt genetic-code change in Coeloplanidae. In agreement with this result, Pett and Lavrov [21] have shown the presence of a mt-TrpRS in the transcriptome of *V. multiformis*. Surprisingly, this enzyme was not detected in the transcriptome of *Coeloplana astericola*. However, this may be the result of a lower transcriptome quality for this species. Unfortunately, the sequencing methods used in this work do not allow us to investigate this issue in *C. loyai* and *C. yulianicorum*.

Previous studies have emphasized the fast mt evolutionary rates of ctenophores when compared to other animal phyla [12, 18]. The long branches of ctenophores in the metazoan tree (Fig. 3) and the high BDn found in inter-order comparisons (Table 1) indicate that not only mt sequence substitutions but also mt gene rearrangements are saturated. Specifically, our increased taxon sampling has revealed that the mt gene order is extremely rearranged among different taxonomic orders of ctenophores, quite variable at the intra-family level and conserved at the intra-genus level, at least in the analysed family Coeloplanidae. Concerning substitution rate, the fast mt evolutionary rate is not limited to a comparison between ctenophores and other animals but also among ctenophore orders. Indeed, the long branch of the *Mnemiopsis* and *Pleurobrachia* cluster compared to the Platyctenida branches (Fig. 3) suggests that the mt genome of ctenophores or its separate genes might present a good marker to investigate ctenophore relationships mainly at lower taxonomic levels, such as at the intra-family or species level (see Alamaru et al. [23]).

The high divergence of ctenophore mt sequences has also complicated their annotation. Our new sequences enabled comparative gene annotations that revealed errors in the original annotation of the *Pleurobrachia* mt genome [18] in gene boundary delimitation or in gene definition, as well as in the identification of missing genes (Additional file 1). However, sequence comparisons did not enable us to identify the *atp8* gene. The *atp8* is a canonical animal mitochondrial gene often overlooked, since it is rather short and poorly conserved. For example, the *atp8* gene was originally believed to be absent in the mt genomes of the tunicate *Halocynthia roretzi* [24], but was later identified there [25]. The presence of URFs in all ctenophore mt sequences suggests that the *atp8* gene might still be encoded in their mt genome. Moreover, unlike the *atp6* gene, the *atp8* has not been identified in the nuclear genome for either *Pleurobrachia* [18] or *Mnemiopsis* [12]. However, the URFs identified in the ctenophore mt genomes neither demonstrate similarity among themselves nor to any known *atp8*. Additional ctenophore mt sequences are therefore needed in order to characterize the *atp8* gene in this phylum.

The ctenophore rRNA genes were found to be about half the size of classical animal mt rRNAs and to share only a few of the helix structures identified in *Mnemiopsis* [12]. Surprisingly, the *rns* gene of *Pleurobrachia* was found to include insertions that were not shared by other ctenophore species. Since the order Cydippida appears well nested within Ctenophora in phylogenetic trees based on nuclear genes [14, 17], it is possible that a secondary size reduction has been occurring in members of this group, while the tremendous size reduction of the rRNA is an ancestral trait.

## Conclusions

In conclusion, the new Platyctenida mt sequences obtained in this work has enabled us to assess for the first time the mt evolutionary rates among and within ctenophore orders. Given their small and compact genomes, Ctenophora could constitute good models in the search to understand the mechanisms leading to variation in mt gene order.

## Methods

### DNA extractions and mt genome sequencing

The benthic ctenophore specimens used in this study (*C. loyai* sample 2013/9; *C. yulianicorum* sample 2002/6; *V. multiformis* sample 2007/1) were collected in the Gulf of Aqaba (29°30′ N, 34°56′ E) in 2012–2013 under permit 2010/37891 from the Israel Nature and National Parks Authority [26].

Genomic DNA (gDNA) extractions were performed with the Qiagen Blood & Tissue kit (Qiagen, Venlo, Netherlands) following the manufacturer’s instructions and using a single individual for each species. To reduce the sequencing cost, we followed the approach of Rubinstein et al. [27] and obtained complete mt sequence from sequencing mixed gDNA samples of several species without barcode. Specifically, the gDNA of the three Coeloplanidae species was mixed with DNA of non-ctenophore species (tunicates) that had participated in other studies [28]. To avoid the assembly of chimera sequences during the assembly step, the two *Coeloplana* species were sequenced as part of a different mix. Library construction and sequencing was performed by the Technion Genome Center (Haifa, Israel). The sequencing of *V. multiformis* and *C. yulianicorum* was performed on an Illumina HiSeq 2000 sequencer with paired reads 100 bp long. The sequencing of *C. loyai* was performed separately on an Illumina MiSeq with paired reads 250 bp long.

The program Cutadapt v1.10 [29] was used to trim reads from both 5′ and 3′ adapters. Rubinstein et al. [27] have shown that genomic assemblers can fail to assemble complete mitochondrial contigs from mixed gDNA samples. Consequently, following Rubinstein et al. [27], reads were assembled with the transcriptome assembler SOAPdenovo-Trans (Release 1.03, 07-25-2013) [30]. Blastn searches were conducted to identify mitogenome contigs using the *cox1* sequence of *Coeloplana* sp. SHL-2011 and *Coeloplana bocki* [31] as query. The mt genome of *V. multiformis* was assembled on a single mt contig. In contrast, the mt genomes of the two *Coeloplana* species were initially each assembled on several contigs (8 for *C. yulianicorum* and 3 for *C. loyai*). Therefore, for these two species tBlastx searches were performed using the mt genome of *Mnemiopsis leidyi* as query [12] with the aim of identifying additional mt contigs. The mt contigs thereby identified were then elongated by read mapping: setting a minimum overlap of 25 bp between a read and a mt contig, we were able to extend a mt contig by at least 75 bp at each round of read mapping, since the single reads are at least 100 bp long. Several rounds of read mapping allowed us to elongate the mt contigs by ~ 500 bp on each end, and Blastn searches were then conducted to identify the contigs whose ends matched.

Once the circular structure of each mt chromosome had been determined, a last paired-read mapping analysis was performed for each species with Geneious Pro (Version 6.1.7, Biomatters, Auckland, New Zealand). This analysis allowed us to confirm the circular organization of the mt genome of each species as well as to verify manually the quality of the assembly for each base of the sequence. The mean coverages of the assembled genomes were 530 (standard deviation [SD] 190; range: 67–901), 11 (SD 4; range: 2–27) and 81 (SD 26; range: 22–160) for *C. loyai*, *C. yulianicorum* and *V. multiformis*, respectively.

The *cox1* sequence of each mt genome was compared to the sequence obtained by Alamaru et al. [23] to confirm that the correct species had been amplified.

### Mt. genome annotation

A preliminary annotation of the mitochondrial sequences was performed with the web server MITOS (revision 656) [10]. The MITOS annotation, however, contained several errors (e.g., absence of genes such as *nad2* and *nad3*; miss-annotated genes such as *atp6*). Consequently, all ORFs detected in the *Coeloplana* and *Vallicula* mt genomes were aligned with the protein genes annotated in the *Mnemiopsis* and *Pleurobrachia* mt genomes. Start and stop codon positions were chosen based on two criteria: minimization of gene overlap and maximization of ctenophore sequence similarity [27]. URFs longer than 100 bp that encompassed putative transmembrane-domain were also annotated. Transmembrane helices were detected in protein sequences with the TMHMM Server v. 2.0 [32] using default settings.

rRNA genes were identified using covariance models as implemented in INFERNAL 1.1 [33]. Specifically, the programs Cmbuild and Cmcalibrate were used with default settings to construct covariance models based on the secondary structure predicted for the *rnl* and *rns* genes of *M. leidyi* [12]. Using these models (one for each rRNA), the program Cmsearch was run with the parameters: -g --notrunc --smxsize 3000 -E 10.000000. The RNA regions detected in each species were then aligned and the start and end of each rRNA gene were defined based on sequence similarity.

tRNA genes were searched with the programs tRNAscan [34], ARWEN [35] and MITOS (which implements MITFI) [10].

Base compositions were computed with Geneious (version R6.1). Potential modifications of the mold genetic code were investigated both manually and with the webserver GenDecoder v.1.6 [20] using default parameters.

Perfect repeats having a repeat unit longer than 20 bp were searched with the REPFIND webserver (http://zlab.bu.edu/repfind/form.html) [36] under default settings with and without the option "Filter low complexity sequence".

### Genome rearrangement

Pairwise breakpoint distances (BD) were computed between each pair of ctenophore mt genomes using the web server CREx [37]. Normalized breakpoint distances (BDn) were obtained by dividing the breakpoint distance by 13 (i.e., the number of genes shared between the ctenophores mt genomes) [38].

### Phylogenetic analysis

Mitochondrial protein coding sequences for representatives of the animal diversity, together with fungi and choanoflagellate outgroups, were downloaded from the NCBI organelle genome resource (https://www.ncbi.nlm.nih.gov/genome/organelle/). Our dataset comprises 20 Cnidaria species, 10 Porifera, 4 Placozoa and 14 Bilateria, plus 6 outgroups.

Translated sequences of *cob*, *cox1–3*, *nad1*, *nad3* and *nad5* (the most conserved ctenophore genes) were aligned at the amino acid level using MAFFT (version 7) [39] under the L-INS-i refinement strategy. The web server Guidance 2 [40] was used to remove positions with a low confidence score (i.e., below 0.93), as well as positions present in less than 25% of the species. The sequence alignments are provided in Additional file 7.

Phylogenetic inference was conducted using PhyloBayes v3.3b. We used the CAT + GTR + Γ mixture model for the Bayesian analysis. Bayesian analyses were conducted with three chains run for 90,000 cycles each and sampled every 10 cycles, with the first 2000 trees discarded as burn-in. The maximum and average differences, observed at the end of the run were 0.09 and 0.0053, respectively. Similarly, we verified that the effsize and rel_diff of all parameters were higher than 50 and lower or equal to 0.3, respectively, which indicates a correct chain convergence.

## Additional files

Additional file 1: Comparison between annotation of the mt genome of *Pleurobrachia bachei* (accession JN392469) [18] and the re-annotation performed in the current work. (PPT 224 kb)

Additional file 2: Alignment of the 5′ region of the *cox1* gene among Ctenophora. Figure, illustrating the difference in the 5′ region of the *cox1* gene between Kohn et al. [18] annotation of the *Pleurobrachia* genome and the amended annotation performed in the current work. (DOCX 15 kb)

Additional file 3: Reannotation of the mt genome of *Pleurobrachia bachei*. Flatfile in Genbank format. (TXT 26 kb)

Additional file 4: Codon usage of Ctenophora species. (XLSX 29 kb)

Additional file 5: Alignment of the *rnl* sequences of ctenophores. (DOCX 937 kb)

Additional file 6: Alignment of the *rns* sequences of ctenophores. (DOCX 584 kb)

Additional file 7: Sequence alignment file used in the phylogenetic analyses. DNA sequence alignment, in Nexus format, used to reconstruct the phylogenetic tree present in Fig. 3. (NEX 193 kb)

## Abbreviations

aaRS: Aminoacyl tRNA synthetase
*atp6/8*: ATPase subunit 6/8 gene
BD: Pairwise breakpoint distance
BDn: Normalized breakpoint distance
bp: Base pair
*cob*: Cytochrome *b* gene
*cox1–3*: Cytochrome oxidase 1–3 gene
gDNA: Genomic DNA
kb: Kilobase
mt: Mitochondrial
*nad1–6*: NADH dehydrogenase subunit 1–6 gene
NC: Non-coding region
NCBI: National Center for Biotechnology Information
PP: Posterior probability
*rnl*: Large subunit ribosomal RNA
*rns*: Small subunit ribosomal RNA
rRNAs: Ribosomal ribonucleic acid
SD: Standard deviation
tRNA: Transfer RNA acid
URF: Unidentified reading frames
