# Supplementary material for: Extensive mitochondrial gene rearrangements in Ctenophora: insights from benthic Platyctenida
Source: BMC Evol Biol. 2018 Apr 27;18:65. doi: 10.1186/s12862-018-1186-1 (PMC5924465; doi:10.1186/s12862-018-1186-1)
Supplement: Supplementary file 1 — Comparison between annotation of the mt genome of Pleurobrachia bachei (accession JN392469) [18] and the re-annotation performed in the current work. (PPT 224 kb) [file 12862_2018_1186_MOESM1_ESM.ppt]

## Slide 1
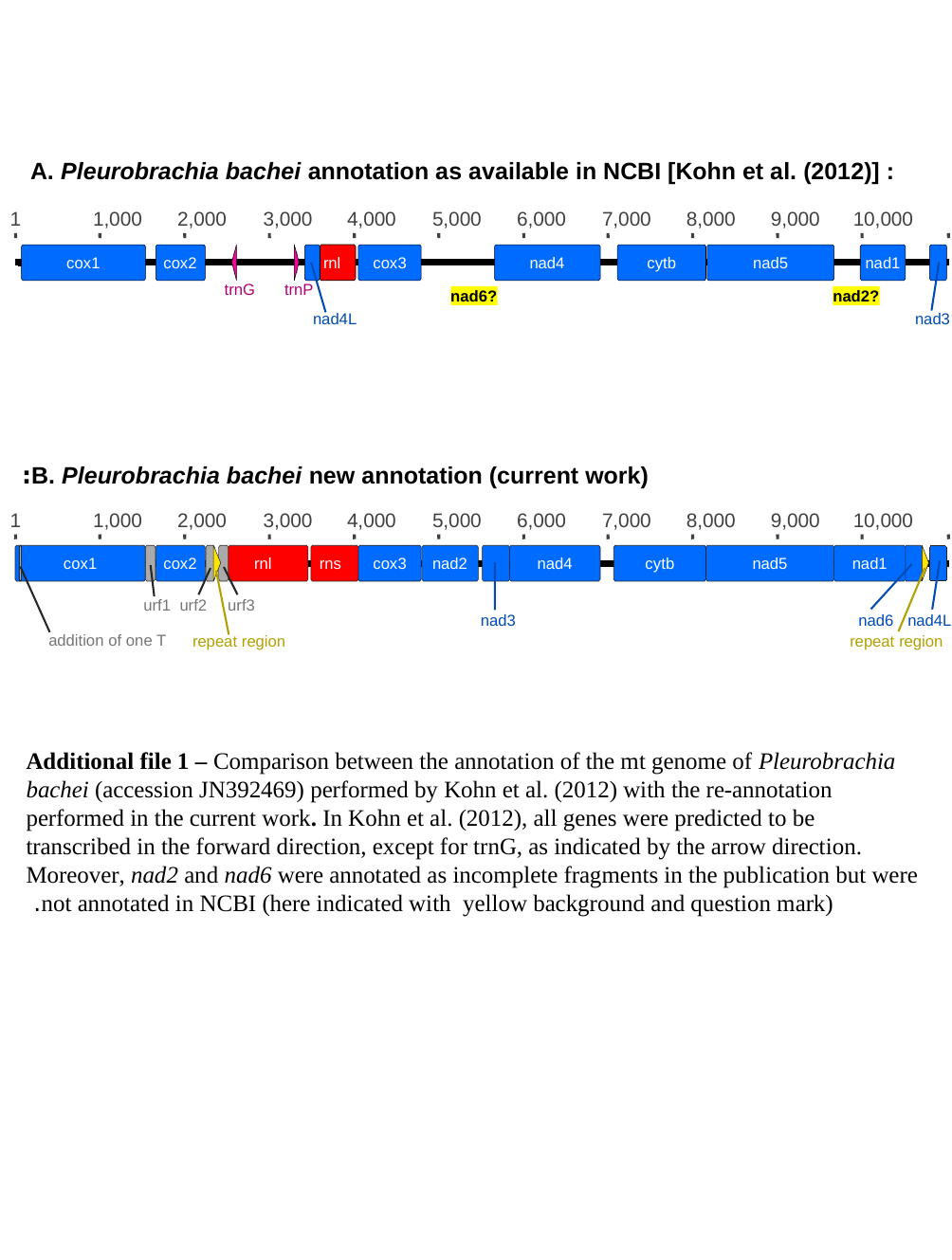

A. Pleurobrachia bachei annotation as available in NCBI [Kohn et al. (2012)] :
1
1,000
2,000
3,000
4,000
5,000
6,000
7,000
8,000
9,000
10,000
cox1
cox2
rnl
cox3
nad4
cytb
nad5
nad1
trnG
trnP
nad6?
nad2?
nad4L
nad3
B. Pleurobrachia bachei new annotation (current work):
1
1,000
2,000
3,000
4,000
5,000
6,000
7,000
8,000
9,000
10,000
cox1
cox2
rnl
rns
cox3
nad2
nad4
cytb
nad5
nad1
urf1
urf2
urf3
nad3
nad6
nad4L
addition of one T
repeat region
repeat region
Additional file 1 – Comparison between the annotation of the mt genome of Pleurobrachia bachei (accession JN392469) performed by Kohn et al. (2012) with the re-annotation performed in the current work. In Kohn et al. (2012), all genes were predicted to be transcribed in the forward direction, except for trnG, as indicated by the arrow direction. Moreover, nad2 and nad6 were annotated as incomplete fragments in the publication but were not annotated in NCBI (here indicated with yellow background and question mark).
