# Supplementary material for: Extensive mitochondrial gene rearrangements in Ctenophora: insights from benthic Platyctenida
Source: BMC Evol Biol. 2018 Apr 27;18:65. doi: 10.1186/s12862-018-1186-1 (PMC5924465; doi:10.1186/s12862-018-1186-1)
Supplement: Supplementary file 2 — Alignment of the 5′ region of the cox1 gene among Ctenophora. Figure, illustrating the difference in the 5′ region of the cox1 gene between Kohn et al. [18] annotation of the Pleurobrachia genome and the amended annotation performed in the current work. (DOCX 15 kb) [file 12862_2018_1186_MOESM2_ESM.docx]

**10 20 30 40 50 60**

**.. |....|....|....|....|....|....|....|....|....|....|....|**

**NC016117 *Mnemiopsis leidyi***  **----MRWLF****STNHKDIASLYFFFSIIMGFCAFFYSFVMRLALVWPFAFIESGIIYLYYVT**

**JN392469 *Pleurobrachia bachei* current work** **MAN-SRWLF****SVFHKDIASLYFFFSIIMGFIGFFYSVIMRLSLSWSYSFITNGVVYLHYVT**

**JN392469 *Pleurobrachia bachei* Kohn et al.**  **--------------------------MGFIGFFYSVIMRLSLSWSYSFITNGVVYLHYVT**

**LN898113 *Coeloplana loyai***  **MFLNSRWLTSTNHKDIGSLYFWFSIFVAFIAFSYSFIIRLSLMWPYSFLIDGNIYNSFVS**

**LN898114 *Coeloplana yulianicorum*** **MFLSSRWLTSTNHKDIGSLYFWFSIFVAFIAFSYSFIIRLSLMWPYSFLVDGNIYNSFVS**

**LN898115 *Vallicula multiformis*** **MFF-FRWFNSTNHKDIGSLYFFFAIFMAFVAFGYSASIRYSLLWPVAFIDFANIYNNAVT**

### Additional file 2 – Alignment of the 5’ region of the *cox1* gene among Ctenophora.

This figure illustrates the difference in the 5’ region of the *cox1* gene between Kohn et al. [[13](#_ENREF_13)] annotation of the *Pleurobrachia* genome and the corrected annotation performed in this work. Pleurobrachia serines encoded by a UGA codon are indicated by a gray background.
