# Supplementary material for: Extensive mitochondrial gene rearrangements in Ctenophora: insights from benthic Platyctenida
Source: BMC Evol Biol. 2018 Apr 27;18:65. doi: 10.1186/s12862-018-1186-1 (PMC5924465; doi:10.1186/s12862-018-1186-1)
Supplement: Supplementary file 5 — Alignment of the rnl sequences of ctenophores. (DOCX 937 kb) [file 12862_2018_1186_MOESM5_ESM.docx]

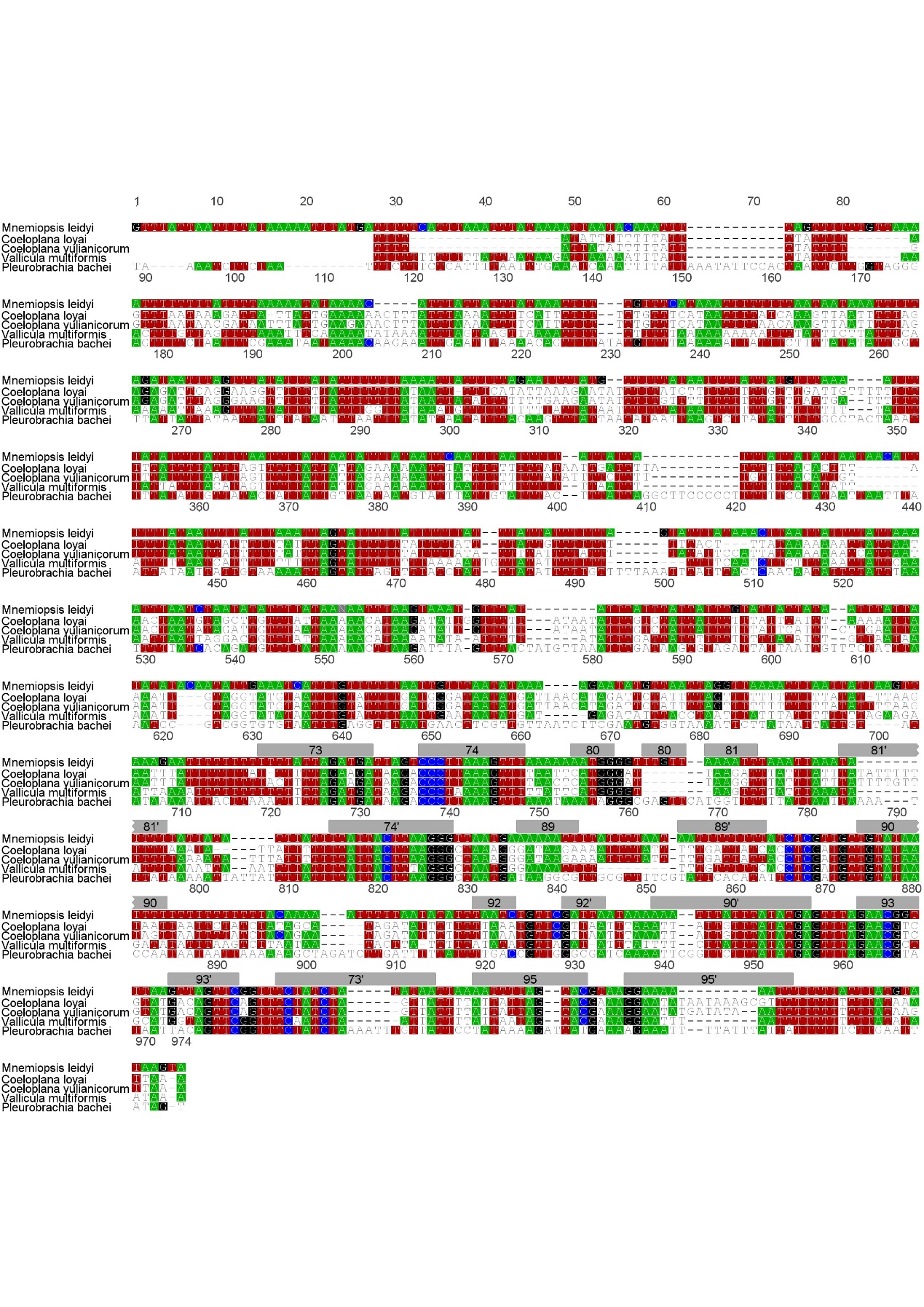


### Additional file 5 – Alignment of the rnl sequences of ctenophores.

The gray boxes indicate the helix identified by Pett et al [10] for *Mnemiopsis leidyi*.
