# Supplementary material for: Extensive mitochondrial gene rearrangements in Ctenophora: insights from benthic Platyctenida
Source: BMC Evol Biol. 2018 Apr 27;18:65. doi: 10.1186/s12862-018-1186-1 (PMC5924465; doi:10.1186/s12862-018-1186-1)
Supplement: Supplementary file 6 — Alignment of the rns sequences of ctenophores. (DOCX 584 kb) [file 12862_2018_1186_MOESM6_ESM.docx]

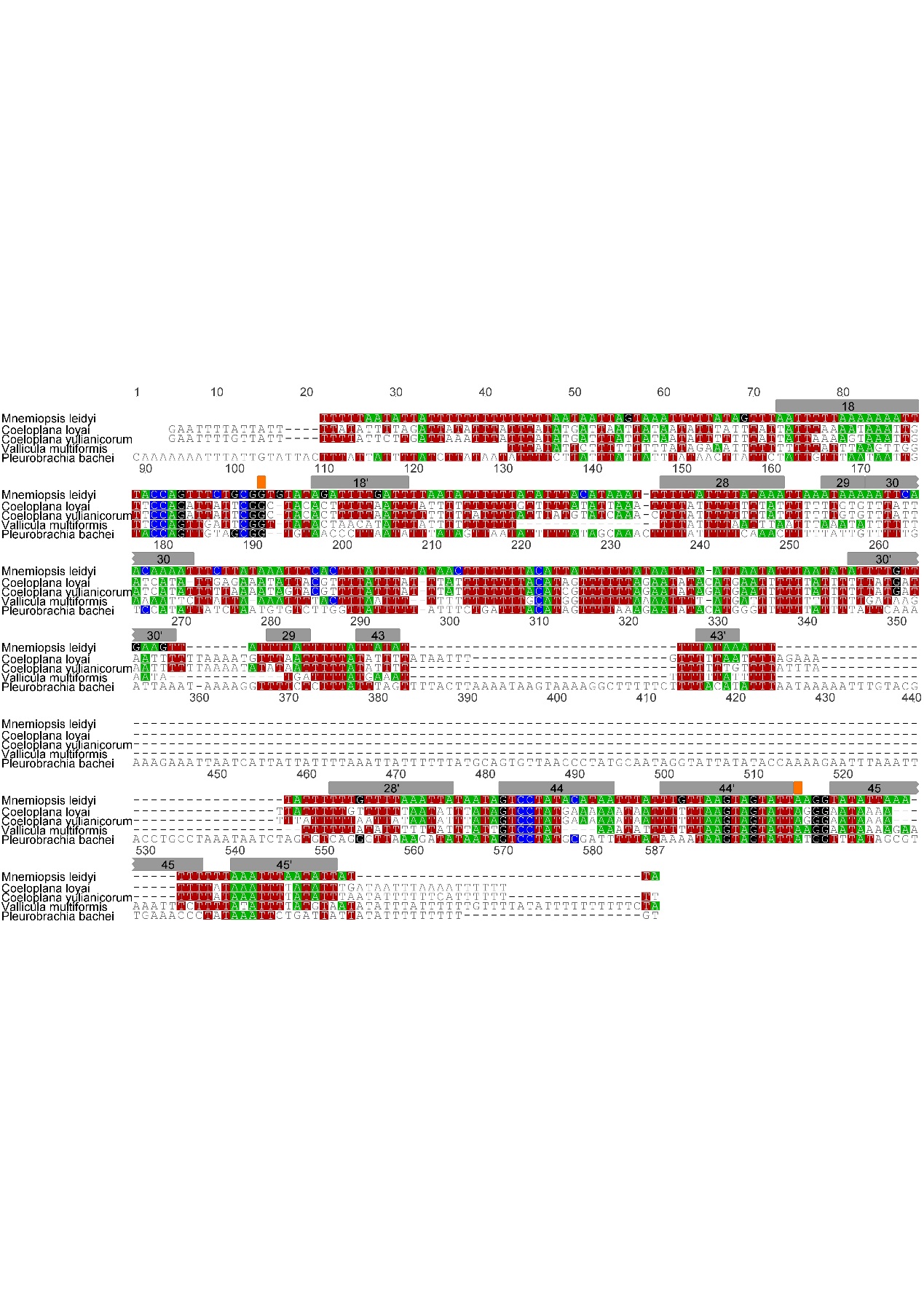


### Additional file 6 – Alignment of the rns sequences of ctenophores.

The gray boxes indicate the helix identified by Pett et al [10] for *Mnemiopsis leidyi*. The orange boxes indicate positions that are critical for the *rns* function.
